# Genome-wide analysis of intracellular pH reveals quantitative control of cell division rate by pH_c _in *Saccharomyces cerevisiae*

**DOI:** 10.1186/gb-2012-13-9-r80

**Published:** 2012-09-26

**Authors:** Rick Orij, Malene L Urbanus, Franco J Vizeacoumar, Guri Giaever, Charles Boone, Corey Nislow, Stanley Brul, Gertien J Smits

**Affiliations:** 1 Molecular Biology and Microbial Food Safety, Swammerdam Institute for Life Sciences, University of Amsterdam, Science Park 904, 1098 XH Amsterdam, the Netherlands; 2 Banting and Best Department of Medical Research, Terrence Donnelly Centre for Cellular and Biomolecular Research, University of Toronto, Toronto, Ontario M5S 3E1, Canada; 3 Department of Molecular Genetics, Terrence Donnelly Centre for Cellular and Biomolecular Research, University of Toronto, Toronto, Ontario M5S 3E1, Canada

## Abstract

**Background:**

Because protonation affects the properties of almost all molecules in cells, cytosolic pH (pH_c_) is usually assumed to be constant. In the model organism yeast, however, pH_c _changes in response to the presence of nutrients and varies during growth. Since small changes in pH_c _can lead to major changes in metabolism, signal transduction, and phenotype, we decided to analyze pH_c _control.

**Results:**

Introducing a pH-sensitive reporter protein into the yeast deletion collection allowed quantitative genome-wide analysis of pH_c _in live, growing yeast cultures. pH_c _is robust towards gene deletion; no single gene mutation led to a pH_c _of more than 0.3 units lower than that of wild type. Correct pH_c _control required not only vacuolar proton pumps, but also strongly relied on mitochondrial function. Additionally, we identified a striking relationship between pH_c _and growth rate. Careful dissection of cause and consequence revealed that pH_c _quantitatively controls growth rate. Detailed analysis of the genetic basis of this control revealed that the adequate signaling of pH_c _depended on inositol polyphosphates, a set of relatively unknown signaling molecules with exquisitely pH sensitive properties.

**Conclusions:**

While pH_c _is a very dynamic parameter in the normal life of yeast, genetically it is a tightly controlled cellular parameter. The coupling of pH_c _to growth rate is even more robust to genetic alteration. Changes in pH_c _control cell division rate in yeast, possibly as a signal. Such a signaling role of pH_c _is probable, and may be central in development and tumorigenesis.

## Background

Cytosolic pH (pH_c_) determines the relative protonation state of all weak acid compounds of the cytosol and affects many, if not all, processes in the cell. It is known to affect redox equilibria [1], metabolic rates and energy storing or generating gradients [2,3], protein interactions [4-7], as well as signal transduction [8-10], and it is an important thermodynamic constraint on metabolic reactions [11]. Furthermore, pH_c _homeostasis is intricately connected with that of other cations, with membrane potentials, and therefore with cellular energy homeostasis [12,13].

Only relatively recently, with the development of green fluorescent protein (GFP)-based pH sensors, has it become possible to study pH in live, unperturbed cells, in an organelle specific fashion [14-18]. The use of this technology is generating increased insight into intracellular pH (pH_i_) control, such as the interaction between plasma membrane and vacuolar proton transport [16,19], and nutrient signaling and pH_i _control [10]. That pH_c _itself directly controls signal transduction and cellular transcriptional responses to environmental conditions was shown recently. A decrease of pH_c _from 7 to 6.8, induced by carbon source depletion, abolishes the interaction of the transcription factor Opi1p with phosphatidic acid through direct protonation of the phosphate headgroup of this membrane phospholipid [7]. Considering the known and emerging importance of this parameter, it is remarkable how little is understood of the genetic basis of its control. We decided to carefully study pH_c _in growing cultures of the yeast deletion collection [20], to determine the genes that are involved in pH_c _control during normal, fermentative growth on glucose. This revealed that pH_c _is controlled by the interplay of cation pumps and the mitochondria, even in fermenting cultures. Additionally, we determined that pH_c _quantitatively controls yeast cell division rate. Remarkably, deletion of 19 genes could abolish this control, suggesting that pH_c _functions as a true signal, transmitting information about external conditions to control cellular decisions.

## Results

### pH_c _is dynamic during growth

Cytosolic pH of yeast cultures is not a static but a highly dynamic parameter. Re-addition of glucose to starved cells leads to a rapid acidification of the cytosol in approximately 30 seconds followed by an alkalinization to neutral pH before growth recommences [18,21,22]. This rapid acidification, which is believed to be the cause of initiation of glycolysis [10,23], is followed by an alkalinization as a result of the activation of the plasma membrane H^+^-ATPase Pma1p [24,25].

**Figure 1:**
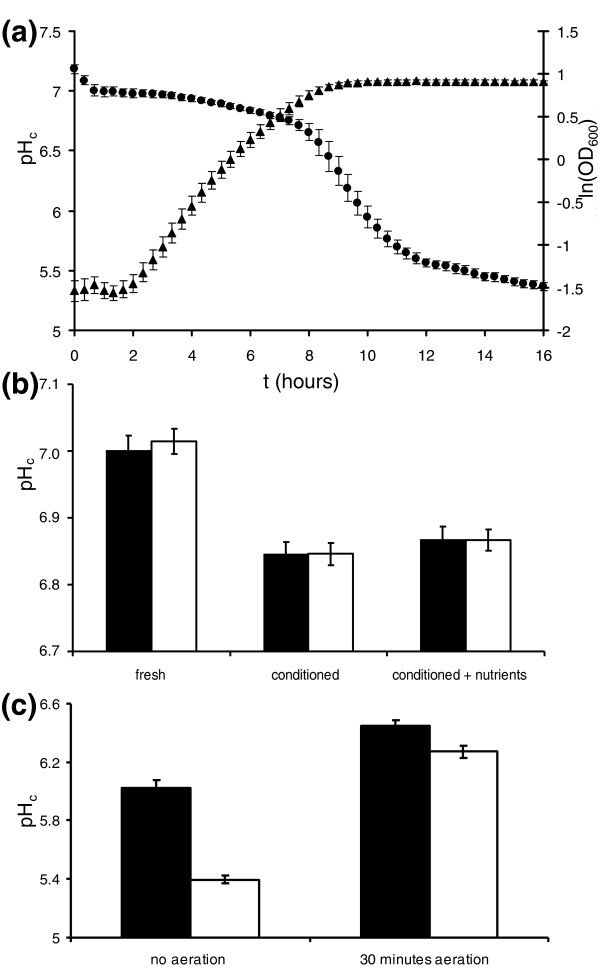
**pH_c _dynamics during growth**. **(a) **Growth (filled triangles) and pH_c _(filled circles) were monitored during growth on glucose in microplates. Data points represent the average of 24 biological replicates, error bars represent standard deviations. **(b) **Medium exchange experiments show that the pH_c _decrease during the growth phase is determined not by the state of the cell, but the environmental conditions generated in the culture medium. Early and late log phase cells were harvested and resuspended in fresh medium, late log medium, and late log medium supplemented with all nutrients. **(c) **CO_2 _causes pH_c _acidification. Strains were grown for 10 hours in either aerobically (closed bars) or anaerobically (open bars). After 10 hours the pH_c _of both strains was determined. Cultures were subsequently aerated for 30 minutes by bubbling air through the culture followed by pH_c _determination.

We assessed pH_c _in growing yeast cultures under batch conditions to reveal that pH_c _is not constant during growth. pH_c _was neutral in the beginning of the exponential growth phase, and then gradually dropped approximately 0.3 pH units in mid- to late exponential phase, before glucose was depleted. Upon glucose depletion pH_c _decreased to 5.5 (Figure 1a). We determined that the gradual reduction of pH_c _from 7 to 6.7 during growth of the culture was a response to changes in the cellular environment, rather than a property of the cultured cells themselves: we took cell and culture supernatant samples from the beginning of the growth phase (where pH_c _was 7.0 and the specific growth rate 0.48 h^-1^) and at the end of the growth phase, just prior to glucose depletion, when the pH_c _and growth rate were decreased. Inoculation of old cells in fresh medium rapidly (within a minute) restored pH_c _to 7.0, while the addition of old, depleted culture supernatant to fresh cells set pH_c _to 6.85 (Figure 1b). Nutrient depletion at the end of the growth phase was not the cause of this intracellular acidification, as supplementation of the conditioned medium with all fresh nutrients did not restore pH_c_. Rather, it appeared that the yeast cells had conditioned the medium such that it now induced a low pH_c_. We determined the concentrations of secreted ethanol and weak organic acids such as succinate, acetate, and pyruvate in the conditioned medium. Addition of the same quantity of these compounds to fresh medium did not lead to a decreased pH_c _(our unpublished data). In contrast, aeration of the conditioned medium relieved its pH_c _lowering effect, strongly suggesting that the main component of the acidifying activity was dissolved CO_2_, acting as a weak acid to acidify the cytoplasm (Figure 1c). Similar acidification occurred even in well shaken Erlenmeyer flask cultures, usually considered properly aerated.

### Identification of pH_c _mutants

**Figure 2:**
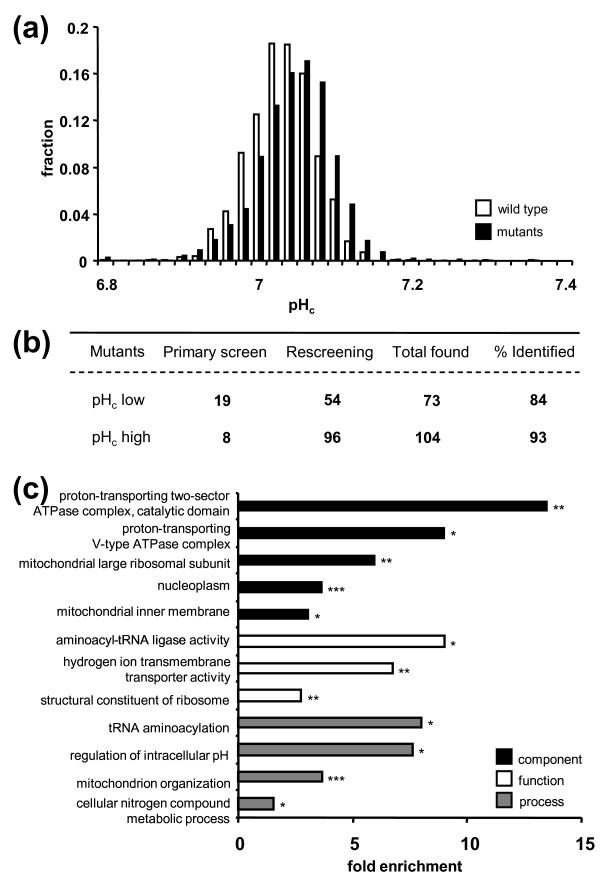
**Genome-wide analysis of pH_c _in growing yeast mutant cultures**. **(a) **Distribution of average pH_c _values of 1,068 wild-type and 4,208 deletion mutant strains in two biological replicate screenings of exponentially growing cultures. **(b) **pH_c _mutants identified in primary and secondary screens. Significantly deviating mutants (*P*-value < 0.01) were identified after six biological replicate determinations of pH_c _in cultures 4 hours after initiation of growth on glucose. **(c) **Hypergeometric enrichment of Gene Ontologies in pH_c _mutants. Fold enrichment is defined as the number of mutants in a given ontology in the pH_c _mutant dataset relative to the number of mutants in that ontology in the screened background dataset (4,740 mutants). *P-value < 0.05, **P-value < 0.01, ***P-value < 0.001.

To understand the genetic basis of the control of this central cellular parameter, we performed a genome-wide analysis of pH_c _in growing cultures of the deletion mutant array (DMA) [20]. The pH-sensitive GFP ratiometric pHluorin [17,18] was introduced in 4,308 haploid mutants of the DMA using synthetic genetic array (SGA) methodology [26]. All mutants were screened in duplicate during mid-exponential phase in microplates at 30°C in medium buffered at pH 5.0. Candidates were selected based on their deviation from the parent pH_c _of 7.08 ± 0.05 (measured in 2,088 biological replicates; Figure 2a). These candidates, along with 432 slow growing mutants that were separately arrayed because they are usually lost during the growth and selection steps of the SGA procedure, were analyzed in six additional biological replicates to obtain statistically valid (*P *< 0.01) hits (Additional File 1, Table S1). Out of a total of 4,740 mutants screened, we identified 177 mutants with significantly high (104) or low (73) pH_c_. All significant mutants were microscopically evaluated for aberrant GFP distribution. No indications of aggregation or cell lysis were found. The pH_c _of these mutant strains was never more than 0.3 pH units below or 0.5 pH units above parent pH_c_. The re-screening of the candidate hits allowed for an accurate false negative estimation (Figure 2b): we applied the criteria for the selection of candidates from the original duplicate screening to the separately arrayed slow grower array, and used this to determine the false negative rate in the initial screening of the 4,308 DMA mutants. This revealed that we successfully identified 86% of the genes with low pH_c _and 95% of the genes with high pH_c_.

We failed to identify several mutants that we expected to have an altered pHc. We separately tested several of these, among which were *trk1, trk2, nha1, nhx1*. In our analysis, cells growing on defined media with glucose available, these mutants did not have a significantly deviating pH_c_. We have shown before [7] that, in such conditions, a *PMA1 *hypomorph *ygl007c *and a *trk1 *deletion strain do not have a pH_c _phenotype, and that such a phenotype only becomes apparent when the cells are challenged with low external pH. At an external pH (pH_ex_) of 3.0, *trk1, trk2, nha1 *and *nhx1 *had pH_c _values of 6.93 ± 0.02, 7.02 ± 0.06, 6.99 ± 0.01 and 7.08 ± 0.10, respectively (with average Z-values of -2.77, -0.99, -1.32 and 0.02), which indicates a significant pH_c _deviation for *trk1 *only. This suggests that, although the gene products have well defined functions in cation homeostasis, deletion of these genes when sufficient cellular energy can be generated does not cause a pH_c _decrease.

### Profiling of mutants with aberrant pH_c _under additional stress conditions reveals involvement of vacuole and mitochondria in pH_c _control

**Figure 3:**
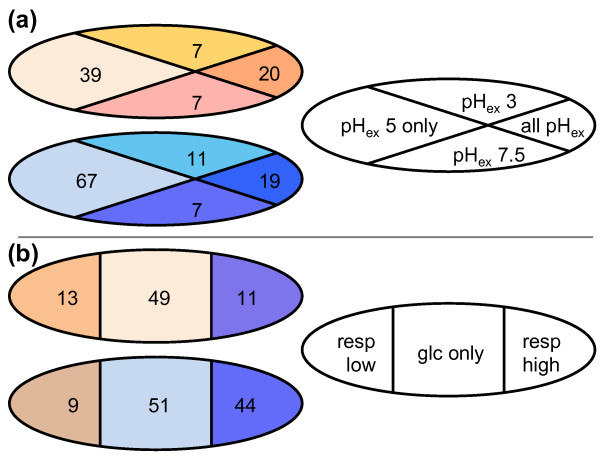
**Profiling of mutants with aberrant pH_c_**. **(a, b) **Venn diagrams of mutants with significantly low pH_c _(top) or significantly high pH_c _(bottom) during growth at the normal pH_ex _of 5.0 and during growth at extreme pH_ex _values of 3.0 and 7.5 (wild-type pH_c _of 7.08 ± 0.03 and 7.20 ± 0.15, respectively) (a), or with significantly low pH_c _(top) or significantly high pH_c _(bottom) during growth in medium with 2% glucose, upon growth on 2% ethanol and 2% glycerol as respiratory carbon source (wild-type pH_c _of 6.93 ± 0.06) (b).

The pH_c _mutants were analyzed for functional enrichment based on their gene ontology terms (Figure 2c). As expected, functional groups related to proton and cation homeostasis were strongly enriched, including vacuolar proton pumping [16]. The genes we identified in these functional groups are required to maintain pH_c_, and we expected, therefore, that these and our other pH_c _mutants would have difficulty to control pH_c _during growth in media conditions that were close to the lower and upper tolerance external pH (pH_ex_) limits for yeast growth. Most *Saccharomyces *strains will grow at pH_ex _values of 2.5 to 8.5, and the kinetics of growth and fermentation are not affected between pH 3.5 and 6.0 because of the tight control of intracellular pH [27]. We decided upon media pH values of 3.0 and 7.5, which induce considerable stress, yet allow for significant cell growth and pHluorin expression. Wild-type pH_c _is unaffected by cultivation at a pH_ex _of 3.0 compared to a pH_ex _of 5.0, and slightly increased during cultivation at a pH_ex _of 7.5 (Additional file 1). We identified 20 mutants that had a low pH_c _at all pH_ex _conditions, and 19 that had a consistently high pH_c _(Figure 3a; Additional file 1), and found that these 39 were enriched for global growth-related functions such as RNA polymerase II-mediated transcription and mitochondrial translation. Remarkably, we did not find a clear indication that loss of proton homeostasis mechanisms led to a pH_c _phenotype that became much more sensitive to pH_ex _changes; if we define a pH-fragile mutant as one that has a high pH_c _at a pH_ex _of 7.5, but a low pH_c _at a pH_ex _of 3.0, we could identify only four mutants (*rpl31a*Δ, *gpm2*Δ, *pet111*Δ, and *erg28*Δ) that had lost the capacity to maintain pH_c _with respect to pH_ex_, which is less than could be expected by coincidence.

Mitochondrial proton pumping functions, as well as functions related to mitochondrial biogenesis and inheritance, were strongly enriched, despite the fact that respiration is not required for energy generation during fermentative growth on glucose. To see if this mitochondrial role in pH_c _homeostasis is related to the role of the mitochondria in energy generation, we assessed the pH_c _of all pH_c _mutants during respiratory growth in glycerol- and ethanol-containing medium (Figure 3b). The average parent pH_c _on respiratory medium was reduced by 0.27 pH units, and similarly almost all mutants tested had a lower pH_c _on respiratory medium. However, only 13 of the 73 strains with a significantly low pH_c _on glucose were also significantly low during respiratory growth, when compared to wild type. Eleven of these (*msf1*Δ, *aep3*Δ, *pet100*Δ, *atp7*Δ, *mss116*Δ, *mtg2*Δ, *mnn9*Δ, *plc1*Δ, *dep1*Δ, *rpl21a*Δ, and *snf2*Δ) had extremely low growth rates on glycerol/ethanol medium, and were indeed previously shown to have severely reduced growth on respiratory media or to be respiratory growth deficient [28-30]. Only *vps66*Δ and *kre28*Δ had low pH_c _on respiratory and fermentative carbon sources but were able to grow relatively normally on respiratory media compared to fermentative conditions. The other 60 strains with low pH_c _during fermentative growth had normal or even high pH_c _during respiratory growth. This apparent rescue of most low pH_c _phenotypes shows that pH_c _regulation in fermenting cells is very different from that in respiring cells, as suggested before [18]. Together, our data establish the major importance of the vacuole and mitochondria in pH control, and reveal these organelles as central controllers of cytosolic ion concentrations [16,19,31,32].

### pH_c _controls growth rate

**Figure 4:**
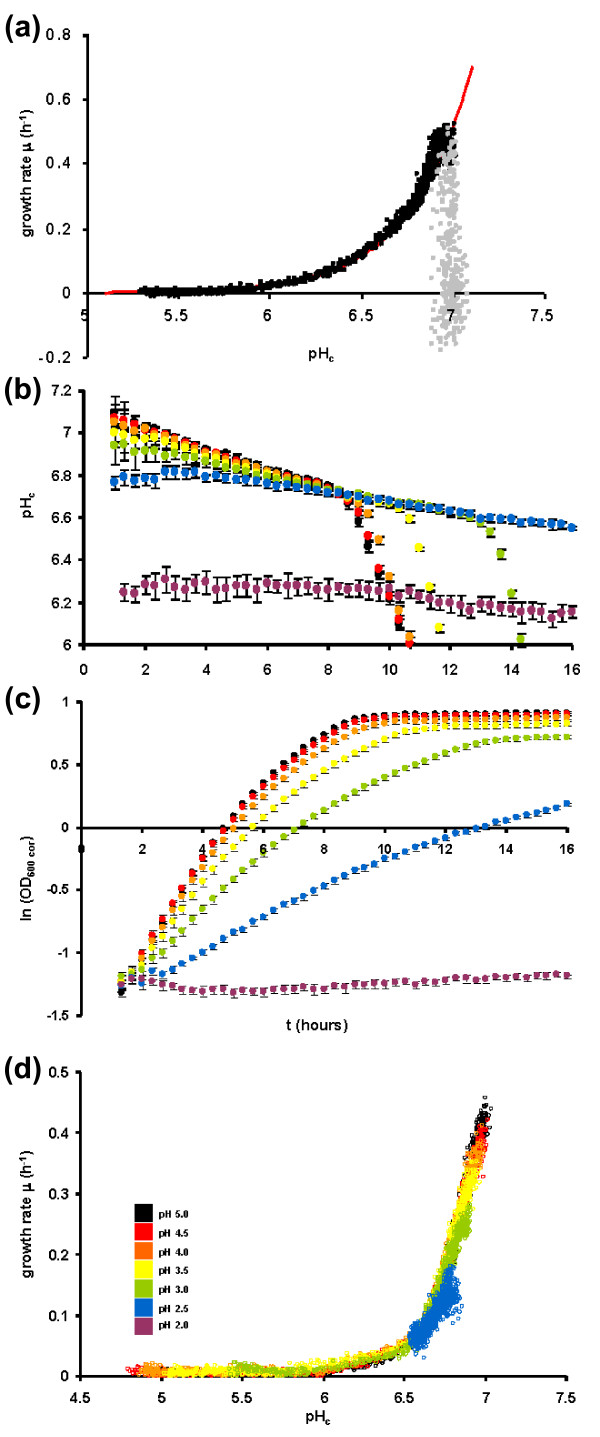
**Cause and effect in the relationship between pH_c _and growth rate**. **(a) **Wild-type pH_c _plotted against growth rate μ. Dots represent the time course data of 24 independent biological replicates of full 16 hour growth curves. The data were fit to an exponential dependence of μ on pH_c _(Additional file 1). Grey dots represent points from the first 1.5 hours after inoculation into fresh medium. **(b-d) **Titration of pH_c _sets growth rate. Strain *pma1-007 *was cultivated in unbuffered, pH_ex _controlled batch fermentors, and titrated to pH_ex _values indicated using strong acid (HCl). Figures represent pH_c _(b), growth (c), and pH_c _plotted against growth rate μ (d). Data points represent the average of 12 technical replicates, error bars represent standard deviations.

Because many growth-related functions were enriched in the deviant pH_c _mutants, we decided to directly analyze the relationship of pH_c _and growth rate in detail. We assessed the relation between growth rate and pH_c _in the parent strain and found a strong correlation between pH_c _and growth rate (μ) over all time points of the experiment after the cells had resumed growth, which could be recapitulated as a linear relation between log(μ) and pH_c _(R^2 ^= 0.99 over all the time points between t = 2 h and t = 16 h from 24 biological replicates) (Figure 4a). Even after glucose depletion (around 8 hours cultivation time in this experiment), when the growth rate is gradually reduced to 0 and the pH is reduced to approximately 5.5, the relationship of pH_c _and growth rate is maintained. Only the time points before t = 2 h (grey dots in Figure 4a) do not fit the relationship but cluster around pH 7,0, because pH_c _already increased upon re-inoculation in fresh medium before growth was observable. The strong correlation of two such central parameters of cellular functioning raises the question if pH_c _might determine growth rate, growth rate pH_c_, or whether there is a process that controls both. To establish causality we first note that effects on pH_c _are rapid, and precede alterations of growth rate. First, at the beginning of growth, pH_c _already increases to 7.0 before growth of the culture starts (Figures 1 and 4a). Second, we established that the gradual reduction of pH_c _from 7 to 6.7 during growth of the culture is rapidly induced by the cellular environment at those times, rather than a property of the cells themselves (Figure 1b). The rate at which the pH_c _dropped when cells were exposed to this conditioned medium suggests that the pH_c _drop precedes the drop in growth rate. The hypothesis that growth rate controls pH_c _is also contradicted by the observation that most slow growing mutants (276 out of 432 tested) had a normal pH_c _(Additional file 2). Together, these data render it highly unlikely that growth rate controls pH_c_.

To positively demonstrate that pH_c _dictates growth rate, we postulated that it should then be possible to titrate pH_c_, which should allow us to set the growth rate to the values predicted by the quantitative relationship previously determined (Figure 4a). In wild-type strains, the plasma membrane ATPase ensures pH_c _control in different pH_ex _conditions [18,33], and titration of pH_ex _leads to only minute changes in pH_c _(Figure S1 in Additional file 3). However, in a mutant with a hypomorphic allele of *PMA1 *(*pma1-007*), with approximately 50% reduced plasma membrane ATPase levels and activity [7,34], acidification of the culture medium with HCl from pH 5.0 to pH 2.0 resulted in a pH_c _decrease from 7.0 to 6.2 (Figure 4b), while nutrient abundance is not affected. Cultivation in these conditions revealed that growth was also progressively decreased (Figure 4c). Direct comparison of growth rate and pH_c _revealed that the growth rate was decreased exactly to the extent expected from the lowering of pH_c _(Figure 4d), thus showing that pH_c _determines the growth rate.

### pH_c _appears to have a signaling role

**Figure 5:**
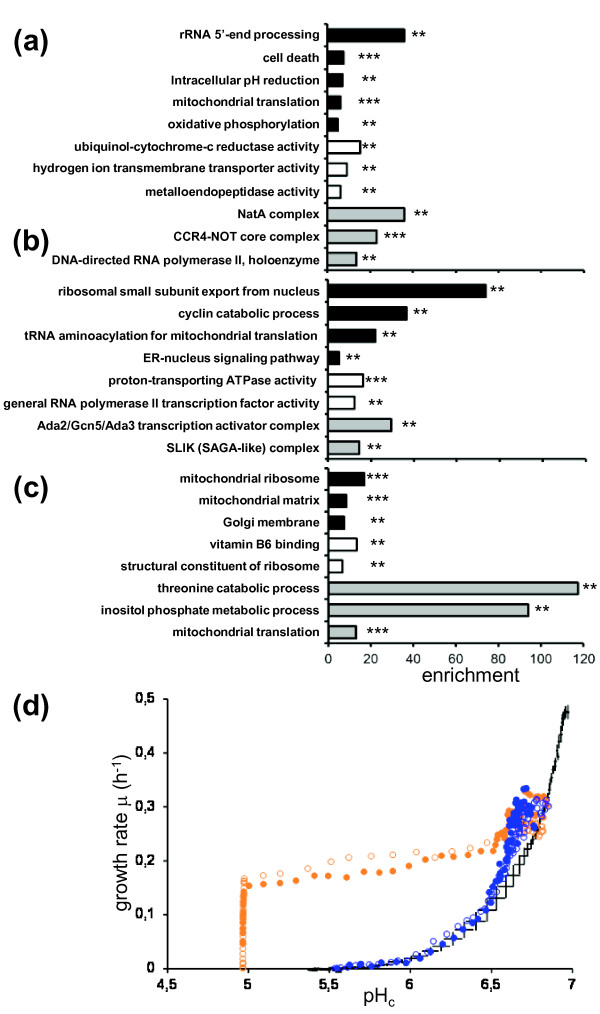
**Mutant pH_c_-growth rate relationship**. **(a**-**c) **Gene ontology enrichment analysis of the wild-type (a), low growth rate (b), and high growth rate (c) cluster. **(d) **In *kcs1Δ *(blue symbols) and *plc1Δ *(orange symbols) growth rate is no longer normally restricted by pH_c_. Biological duplicates (open and closed symbols) are shown, compared to wild-type variance of 96 replicates indicated by average ± standard deviation of both pH_c _and growth rate (black crosses).

Now that we have determined that pH_c _controls growth rate, we could analyze the genes that are required for maintaining this relationship. Analysis of all pH_c _mutants with respect to this same pH_c_-growth rate relationship revealed three distinct classes of mutants (Figure 5a; Figure S2 in Additional file 3; Additional file 4). Category I had a pH_c _that significantly deviated from wild type pH_c _during exponential growth, but the normal relationship between pH_c _and growth rate was maintained. Wild-type strains analyzed of course cluster with this category. The genes mutated in this case can be considered upstream of pH_c_, and thus control pH_c_. The effect they have on growth rate is through their effect on pH_c _only. This category contained the majority of the mutants identified in our screen (92 out of 173 mutant strains). Most of the cation and pH homeostasis-related genes identified in the screen fell into this category, as well as many involved in mitochondrial translation and oxidative phosphorylation (Figure 5b). Category II (62 out of 173 mutant strains) had a significantly low growth rate compared to the pH_c_, which suggests that these mutations limit growth rate though mechanisms other than pH_c_. Indeed, many of these mutants had a high pH_c _in our screen (45 out of 62), even while growth rate was reduced compared to wild-type. We selected a set of slow growing mutants with deletions in genes involved in ribosome biogenesis and central carbon metabolism that do not exhibit aberrant pH_c_, and used the same analysis of growth rate/pH_c _relationship, to show that these would fall into the same category (Figure S3 in Additional file 3). Indeed, very general functions are enriched in this cluster, such as polymerase II-mediated transcription, and ATP generating processes (Figure 5c). Any effect of such functions on pH_c _is likely indirect - for instance, because expression of pH regulatory genes is RNA polymerase II mediated [35,36], or proton homeostatic processes depend on ATP [2,3]. Finally, mutants in category III had a low pH_c _and regularly also a low growth rate, but, more importantly, their growth rate was no longer as constricted by this low pH_c _as in the wild type. Only 19 of 173 mutants fall into this category (Table 1), suggesting a coupling that is robust to mutations in single genes. The fact that the deletion of single genes can lead to (partial or full) evasion of the control of pH_c _over growth rate shows that pH_c _is not somehow rate limiting for growth because of the complex integrated effect of protonation of many cellular components. This strongly suggests that pH_c _functions as a true signal that must be transduced. Although again the genes in this category were enriched for basic mitochondrial functions, we also found enrichment for genes involved in signal transduction and inositol pyrophosphate metabolism (Figure 5d). These high-energy metabolites are proposed to be involved in the sensing of a cell's energy status [37]. Interestingly, *kcs1Δ *and *plc1Δ*, involved in inositol pyrophosphate metabolism, both show growth that is no longer limited by pH_c _as it is in the wild type (Figure 5e). For *kcs1Δ*, this entails a growth rate of 0.30 h^-1 ^instead of the expected 0.18 h^-1 ^at a pH_c _of 6.7, and the difference is most apparent at relatively normal pH_c _values. For *plc1Δ *the phenotype is even more striking, with growth rate being more or less constant (although never very high) until pH_c _values of 5, below which growth suddenly collapses. These two mutants are both among the mutants with the lowest pH_c _in the screen (Additional file 1). The pH dependence of the charge of the many phosphates on inositol poly- or pyrophosphates renders these metabolites exquisitely suitable for pH_c _sensing. Three other mutants in the inositol pyrophosphate synthesis pathway, *vip1Δ *and *ipk1Δ *(deletion of genes encoding enzymes responsible for pyrophosphorylation of inositol hexakisphosphate (InsP6) at the 1-P or 3-P position) and *ddp1Δ *(deletion of the gene encoding the phosphatase that removes the pyrophosphates) [38,39], did not affect pH_c _or pH_c _signaling to growth rate (Figure S4 in Additional file 3).

**Table 1:** Mutations that reduce growth control by pH_c_

Category	Gene	ORF	**Absolute pH**_**c**_^**a**^	GO process
Inositol polyphosphates	*PLC1*	*YPL268W*	6.92 (0.035)	Inositol phosphate biosynthetic process
	*KCS1*	*YDR017C*	6.76 (0.047)	Inositol phosphate biosynthetic process
				
Signal transduction	*LCB5*	*YLR260W*	6.99 (0.034)	Calcium-mediated signaling
	*RHO5*	*YNL180C*	7.02 (0.025)	Rho protein signal transduction
				
Lipid synthesis	*CAX4*	*YGR036C*	6.93 (0.057)	Lipid biosynthetic process
	*PDX3*	*YBR035C*	6.99 (0.025)	Fatty acid metabolic process
				
Vesicular transport	*RGP1*	*YDR137W*	6.92 (0.031)	Retrograde transport, endosome to Golgi
	*CLC1*	*YGR167W*	7.01 (0.049)	Endocytosis
				
Mitochondria	*RSM24*	*YDR175C*	7.01 (0.024)	Mitochondrial translation
	*MRPL27*	*YBR282W*	7.00 (0.019)	Mitochondrial translation
	*MRPL16*	*YBL038W*	6.97 (0.028)	Mitochondrial translation
	*MRPL37*	*YBR268W*	6.98 (0.032)	Mitochondrial translation
	*MRPS5*	*YBR251W*	7.00 (0.027)	Mitochondrial translation
				
Various	*MSS2*	*YDL107W*	7.00 (0.035)	Protein insertion
	*ILV1*	*YER086W*	6.98 (0.035)	Isoleucine biosynthetic process
	*REF2*	*YDR195W*	6.97 (0.072)	mRNA processing
	*CYC8*	*YBR112C*	7.00 (0.032)	Chromatin remodeling
				
Unknown	*AIM44*	*YPL158C*	6.97 (0.024)	Biological process unknown

^a^Wild-type pH average is 7.08 ± 0.05. Mutants were clustered according to pH_c _with respect to growth rate (Materials and methods) and grouped according to functionally related gene ontology (GO) processes.

## Discussion

### Complex control of a simple parameter

It is becoming apparent that pH_c _is controlled not just by simply pumping protons out of the cytosol into the vacuole or across the plasma membrane. The pH of the various compartments is heavily influenced by that of others [16]. Vacuolar ATPases are not only important for maintenance of the low pH in the secretory pathway, but also for control of cytosolic pH. This could be a buffering activity, a transient and rapid mode of removing protons from the cytosol, to be made permanent by true proton efflux. It should be noted, however, that a transient storage of protons in the secretory pathway or the vacuole conserves the energy in the cell as a membrane potential. Most of the components of the vacuolar ATPases have been identified based on the *vma *phenotype, which comprises the inability to grow at high extracellular pH and at high Ca^2+ ^concentrations [40,41]. It is remarkable, therefore, that not all *vma *mutants have a pH_c _phenotype at a pH_ex _of 5.0, and of the ones that do, not all have a pH_c _phenotype at a pH_ex _of 7.5 (2 out of 7). Similarly, we found very little correlation between our screen for pH_c _and a recent screen for mutations leading to deviating vacuolar pH, although conditions were different [42]. This suggests that even if the mechanisms for pH_c _control share components with those for vacuolar pH control, the organelles control their pH independently.

While the role of vacuolar proton pumping in pH_c _homeostasis has been relatively well studied, the important role of the mitochondria is novel. In an analysis of mutations affecting the 'ionome' of yeast, a similar enrichment of both vacuolar and mitochondrial genes was observed [31], suggesting that the homeostasis of cations in general requires the proper function of both organelles. However, this analysis does not distinguish the organelle-specific ion concentrations. A role for mitochondria in pH_c _homeostasis is less surprising if one considers the fact that proton translocation over the mitochondrial inner membrane is the driving force for energy generation in mitochondria. However, in the glucose-rich conditions of our screen the mitochondrial respiratory chain is not required for energy generation in yeast. Still, we identified a large number of mutants in mitochondrial genes in which pH_c _was affected. The genes were classified as part of the proton translocating machinery and the mitochondrial respiratory chain, but also as involved in mitochondrial translation and biogenesis. The same mutations did not necessarily affect pH_c _in respiratory conditions, which suggests that the role of these genes in pH_c _homeostasis is indeed not linked to energy generation.

Clearly, the control of pH_c _in yeast, and in other eukaryotes [32,43] is distributed over various organelles, as is control of organellar pH. Also, the same functions are not responsible for pH_c _control in different conditions. This shows that pH_c _is a seemingly simple outcome of diverse and intricate regulation. This renders pH_c _an excellent signal of cellular status.

### pH_c _as a signal integrating multiple environmental cues to control growth

In yeast it has long been known that pH_c _transiently responds to the addition of nutrients [18,23]. The transient pH_c _reduction upon glucose addition plays a role in the activation of the nutrient signaling, growth-promoting activation of protein kinase A [44]. How exactly pH_c _functions in the activation of protein kinase A is not yet fully understood, but it is clear that a lowered pH_c _promotes the activity of the adenylate cyclase Cyr1p through the activity of Ira2p and Ras2p [45], and possibly also the increase in the affinity of Cyr1p for ATP upon a decrease in pH_c _[46]. Additionally, the gradual decrease of pH_c _upon glucose depletion was shown to lead to inactivation of protein kinase A, through the disassembly of the vacuolar proton ATPases [10,19]. Decrease of pH_c _upon glucose depletion also affects other signal transduction pathways. The activation of inositol biosynthetic gene transcription upon inositol depletion is inhibited in the absence of a proper energy source, through direct control of the activity of the transcriptional inhibitor Opi1p. This inhibitor is recruited to phosphatidic acid in membranes upon inositol depletion, directly binding to the negatively charged phospholipid. Upon pH_c _decrease the phosphate headgroup of the phospholipid is protonated, and Opi1p is released [7,47]. Therefore, as phosphates can be protonated at physiological pH_c _values, and this can affect interactions with proteins, it is very likely that other phosphate groups, such as those on proteins or those on small metabolites, may be protonated and this protonation could then affect recognition by enzymes or effector proteins.

**Figure 6:**
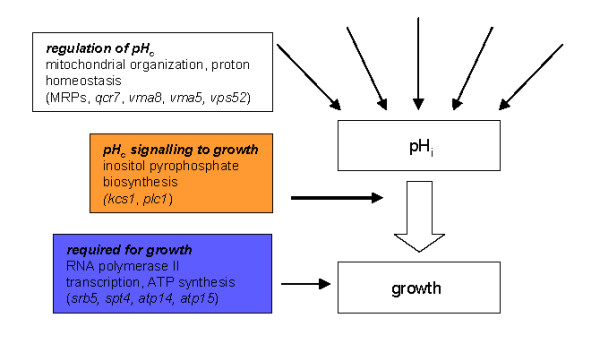
**Schematic model for the regulation of pH_c _and its control of growth rate**.

We identified three different classes of pH_c _mutants with respect to their cell division rate. The three classes are suggestive of different modes of functional interference with the pH_c_-growth rate interdependence (Figure 6). We reasoned that any integrated effect of increased protonation of many cellular compounds that would lead to reduced growth by reduced pH_c _could not possibly be relieved by deletions of single ORFs. The fact, therefore, that strains mutated for a single gene have a higher than expected growth rate at a given pH_c _based on the wild-type pH_c_-growth rate relationship suggests that pH_c _would function as a true second messenger [7,10], providing a signal that is improperly transduced in only a very small number of deletion strains. This last observation reveals that the tight control of growth rate by pH_c _has evolved to become robust towards mutation, suggesting a high importance for cell or population fitness.

There is some evidence that pH_c _varies during the cell cycle [48,49], but others have not been able to confirm this [50]. We show that pH_c _quantitatively controls cell division rate. This may or may not be through cell cycle phase-dependent fluctuations. However, the fact that the changes in pH_c _that we observed are in the range at which phosphate groups can be protonated, and the observation that such protonation can interfere with protein interactions [7], would allow many opportunities for pH_c _interference with cell cycle signaling.

Previously, is has been shown that the Vip1p pyrophosphorylation of InsP6 is important for phosphate regulation of growth [51,52]. Interestingly, they reveal an inhibitory interaction of inositol pyrophosphate with the alternative CDK Pho85p, suggesting that such interactions could control kinase function [39,52]. We now show that Kcs1p-mediated pyrophosphorylation of InsP6 is important for pH_c _signaling to set growth rate. It is tempting to speculate that these inositol pyrophosphate species appear to integrate signals from two major regulatory metabolites to determine growth rate. Indeed, a recent publication shows that inositol pyrophosphates contribute to cellular energy control [53], which is completely in line with the potential role of pH_c _in nutrient status sensing. The authors find that inositol pyrophosphates affect mitochondrial function and fermentation rates to control cellular ATP content. This suggests that pH_c_, inositol pyrophosphates and mitochondria function in an interdependent network to control cell division, resource distribution, and therefore potentially the balance between growth and cellular robustness [53-57].

### The implications of pH_c _as a signal for growth and more

In humans, pH_c _changes have been implicated in tumorigenic transformation and tumor growth and metastasis [58-60]. The reduction of oxygen availability in solid tumors causes a fermentative metabolism, described already in 1927 by Otto Warburg. This change leads to the production of lactate, which causes the acidification of the tumor exterior space and the surrounding tissue. Tumor cells themselves almost invariably overexpress pH homeostatic pumps [61,62], leading to a selective advantage of transformed over non-transformed cells. Interestingly, our data suggest that the acidification of the non-transformed cells might result in a growth suppression, but also that the slightly alkaline tumor cell cytosol might have growth promoting activity. Indeed, it has been suggested that tumor alkalinization might already be a very early event in tumorigenic transformation [60], and an even slightly increased replication rate of the alkalinized cells could benefit the accumulation of mutations promoting the transformation to malignancy.

## Conclusions

Intracellular pH is a determinant of the properties of the molecules that make up living cells, and changes in pH_c _rapidly and reversibly alter these properties. In yeast, pH_c _is highly dynamic. Although intracellular pH is hardly affected by changes in extracellular pH, it is affected by the changes occurring during normal growth on glucose (Figure 1), and decreases as a consequence of CO_2 _produced by cellular metabolism. In respiratory conditions, either after glucose depletion or during growth on glycerol- and ethanol-containing media, pH is much lower than during fermentation.

To understand the genetic basis of pH_c _control during growth on glucose, we expressed the pH-sensitive GFP pHluorin in all strains of the yeast haploid deletion collection. We analyzed pH_c _in liquid cultures of these strains during the exponential growth phase, and identified 177 mutants in which pH_c _deviated significantly from wild type. The genes affecting pH_c _control were strongly enriched for functions in the vacuole, including vacuolar proton pumping, but also for many mitochondrial functions, both in proton pumping and energy generation as well as in mitochondrial translation, biogenesis and inheritance. The fact that the same mutations did not lead to pH_c _alterations in respiratory conditions indicates that pH_c _control is condition dependent.

The most striking functional enrichment of the mutants was in very general growth-related functions. This could imply that pH_c _is reduced as a consequence of reduction in growth rate or as a consequence of the metabolic conditions that lead to a reduced growth rate, but it also opens up the possibility that growth rate is reduced as a consequence of the reduction in pH_c_. We note that while conditions that decrease growth rate also generally reduce pH_c_, mutations that reduce growth rate do not necessarily reduce pH_c_. Also, pH_c _responds much more rapidly to external conditions than growth rate does. This suggests that either environmental conditions affect pH_c _and metabolism that sets growth rate in parallel, or that pH_c _in fact controls growth rate. To distinguish between these two options, we titrated pH_ex _with strong acid in cultures of the plasma membrane ATPase hypomorph *pma1-007 *that were otherwise unaffected in nutrient availability, and thereby reduced pH_c_. This indeed led to a reduction of growth rate, quantitatively matching the relationship between pH_c _and growth rate observed in wild type. This proves that pH_c _directly controls growth rate. A further analysis of the genetic basis of this control shows that it was relaxed in only 19 out of 177 mutants. The fact that pH_c _control can be relaxed demonstrates that pH_c _must function as a signal, whereas the fact that this signal is not properly sensed in only 19 mutants suggests that it has evolved to be robust to mutation, and therefore that it is central and important.

Indeed, there is evidence that similar control takes place in mammalian cells. Although a link between pH control and tumorigenesis has been established, most research in this field focuses on the acidified cell exterior, which appears to affect metastatic propensity [59]. Very little is known about the role of the alkalinization of the cell interior. However, recent literature also suggests that, in mammalian cells too, pH_c _affects signal transduction [8,9] and cell division rate [63-65]. The fact that we now show that cytosolic pH controls growth rate, and functions as a signal to do so, suggests that the relevance of pH_c _for growth control is conserved. How exactly this quantitative signal is controlled, and how it is transmitted to control the cell cycle, remains to be elucidated, but the involvement of the inositol pyrophosphate kinase Kcs1p, and of Plc1p gives promising targets for investigation.

## Materials and methods

### Growth conditions

In all experiments strains were pre-cultured by transfer from solid medium to liquid and incubated overnight (o/n) in round-bottom 96-well microplates (Greiner Bio-One, Kremsmünster, Austria)) stacked in microplate holders (New Brunswick, Enfield, CT, USA) under constant shaking at 30°C in 200 μl low fluorescence synthetic defined monosodium glutamate medium buffered at pH 5.0 with 25 mM sodium citrate. This medium contained 1.7 g yeast nitrogen base without (w/o) ammonium sulfate, w/o folic acid, w/o riboflavin (MP Biochemicals, Santa Ana, CA, USA), 1 g L-glutamic acid sodium salt hydrate (Sigma-Aldrich, St. Louis, MO, USA), 2 g drop-out amino acids supplement powder mixture without uracil (Sigma-Aldrich; Acros Organics, Geel, Belgium; Thermo Fisher Scientific, Waltham, MA, USA), 20 g glucose (Thermo Fisher Scientific) and 7.35 g sodium citrate (Sigma-Aldrich) per liter. For pH titration experiments, strains were cultivated in 500 ml batch fermenters with a steady airflow (500 ml minute^-1^) and stirring rate (600 r.p.m.). The pH of batch fermenter cultures was controlled by titration with 0.2 M KOH using an Applikon ADI 1030 controller (Applikon Biotechnology, Foster City, CA, USA).

### Strain construction

The plasmid pYES-P_ACT1_-pHluorin [18] was introduced into the *MAT**α ***query strain Y7092 and crossed with the *MAT***a **DMA using SGA technology [26], yielding a complete set of mutants expressing pHluorin in the cytosol. Mutants that displayed a slow growing phenotype were set up in a separate array, allowing for longer incubation times on the different SGA media.

### Metabolite analysis

Ethanol, acetate, succinate and pyruvate concentrations in the medium were determined by high pressure liquid chromatography. To this extent, 1.0 ml of sample was quenched with 100 μl of 35% perchloric acid followed by a neutralization with 55 μl of 7 M KOH. Samples were analyzed on a Phenomenex Rezex ROA-Organic Acid H^+ ^column (Phenomenex, Utrecht, The Netherlands) using 7.2 mM H_2_SO_4 _as mobile phase.

### Screening for mutants with deviating pH_c_

Unless stated otherwise, strains were pre-cultured o/n in microplates, diluted 10-fold into fresh medium in CELLSTAR black polystyrene clear-bottom 96-well microplates (Greiner Bio-One) and grown for 4 hours to ensure exponential growth. Plates were transferred to the Automated Cell, Compound and Environment Screening System (ACCESS) [66] and incubated at 30°C under constant shaking until automatically transferred to a Safire^2 ^microplate reader (Tecan, Männedorf, Switzerland). Optical density at 600 nm (OD_600_) was measured followed by two fluorescence measurements at 390 nm and 470 nm excitation, both with 512 nm emission. After background subtraction the ratio of the two fluorescence measurements was compared to a calibration curve to determine pH_c _as described in [18]. The array was screened twice and the 2,136 wild-type measurements were used to determine an average wild-type pH_c _with according standard deviation. For every mutant in the array the average pH was converted to a Z-value defined as the number of standard deviations their measured pH was removed from the average wild-type pH_c _in that replicate. Mutants with a Z-value at least higher than 2 or lower than -2 once and higher than 1 or lower than -1 in the other screening were selected as 'candidate hits' and combined with the slow grower array for additional confirmatory screening. Candidate hits and slow grower arrays were precultured as described and subjected to six rescreening runs where OD_600 _was measured with a SpectraMax Plus384 microplate spectrophotometer (Molecular Devices, Sunnyvale, CA, USA) followed by pHluorin fluorescence measurements using a SpectraMax Gemini XS microtitre plate spectrofluorometer (Molecular Devices). In each of these replicates, mutant pH_c _values were converted to Z-values with respect to average and standard deviation of the wild-type population in that replicate. Significant scores were determined based on a two-tailed *t*-test of these rescreening Z-values compared to the wild-type data (*P *< 0.01). For the analysis of the candidate hits based on the initial two genome-wide screens, for rigidity we did not include the pH_c _values used for identification in the determination of significance. Doing so would have improved *P*-values for all pH_c _mutants identified. For the slow growing mutants, to avoid bias originating from outliers, we performed a second analysis discarding the lowest and highest replicate value for each mutant (Jackknife), and retesting significance. Mutants with *P*-values > 0.1 in this analysis were discarded as significant. Both average Z-values and average pH_c _values are presented. All mutants identified as significant were subjected to microscopical analysis: strains were grown in microplates for at least 2 hours as described above. After washing with phosphate-buffered saline, cells were transferred to agarose-coated glass slides. Images were obtained using a Canon A620 camera (Canon, Tokyo, Japan) on an Axiovert 40 CFL microscope with a Plan Neofluar 100 X/NA 1.3 oil objective (Carl Zeiss, Oberkochen, Germany), using Endow GFP narrow-band excitation filter sets (Chroma Technology, Bellows Falls, VT, USA) for fluorescent images.

### Profiling of mutants with aberrant pH_c _under additional stress conditions

After o/n inoculation in described conditions, cells were transferred to fresh medium buffered at pH 3.0 with 25 mM potassium citrate (Sigma-Aldrich), pH 7.5 with 25 mM MOPS (Sigma-Aldrich) or 2% ethanol and 2% glycerol as the carbon source. These experiments were conducted at least three times. Deviation was determined using *t*-tests comparing to the parent strain.

### Hypergeometric distribution analysis

Enrichment of data sets for gene ontology terms was performed using the Yeast GO-term finder [67].

### Growth curve analysis

After o/n inoculation hits identified in the initial screen were re-inoculated and subsequently monitored for 16 hours by OD_600 _and pH_c _measurements every 10 minutes in a FLUOstar OPTIMA microplate reader (BMG labtech, Ortenberg, Germany). Growth data were corrected for the non-linearity of optical recording at higher cell densities similar to the method in [68], correcting OD_600 _values recorded in the microplate reader (OD_obs_) to reflect linear OD_600 _determinations of the same cultures in a Novaspec II spectrophotometer (Pharmacia LKB Biochrom, Cambridge, UK) (always diluting the cultures to yield measured values between 0.05 and 0.3 OD units) using the formula OD_cor _= 1.1252(OD_obs_)^2 ^+ 0.6808(OD_obs_) - 0.0002. Growth rates of cultures in time were determined by calculating the slope of six consecutive data points (1 hour) of the entire ln(OD_cor_) curve.

### Cluster analysis

For cluster analysis 190 independent 12-hour pH_c_-growth curves of the wild-type strain were used to fit the variables in:

(1) $$\mu =10^{\frac{pH_{c}+a}{b}}$$

where *a *was determined be to -7.24 ± 0.06, and *b *to 0.73 ± 0.08. We used this fit to predict the growth rate at each time point based on the pH_c _measured at that time, for all wild-type and mutant strain time courses. We next determined the biological variance at each time point of the measured μ values around the predicted μ values of the 190 individual wild-type replicates, resulting in a mean and standard deviation of the wild-type divergence from the prediction at each time point. Using this mean and standard deviation, we transformed individual wild-type and mutant curves to Z-values at all time points. *t*-Test analyses of the average mutant Z-values compared to all wild-type replicates for all time points between t = 4 h to t = 9 h was multiplied by a correction factor to eliminate false positives based on *P*-values of wild-type time courses in a same *t*-test. Z-values of mutant and wild-type μ/pH_c _curves, from t = 4 h to t = 9 h, were hierarchically clustered using Euclidean distances with full linkage.

## Abbreviations

DMA: deletion mutant array; GFP: green fluorescent protein; InsP6: inositol hexakisphosphate; OD: optical density; ORF: open reading frame; pH_c_: cytosolic pH; pH_ex_: external pH; SGA: synthetic genetic array.

## Competing interests

The authors declare that they have no competing interests.

## Authors' contributions

RO designed and performed most of the experiments and their analysis, and participated in drafting the manuscript. MLU set up the genome-wide screening. FJV performed the SGA recombination experiments. GG, CB, CN and SB assisted in experimental design, analysis, and drafting of the article. GJS conceived of the study, participated in its design and coordination, performed most data analysis and drafted the manuscript. All authors read and approved the final manuscript.

## Supplementary Material

Additional file 1 **All mutants with deviating pH_c _at various pH_ex_, and during respiratory growth**. Mutants with aberrant pH_c _under the standard condition (glucose, pH 5.0) were subjected to growth in pH 3.0, 7.5, as well as 2% ethanol/2% glycerol pH 5.0. Mutants were pre-grown overnight under standard conditions except for the 2% ethanol/2% glycerol experiment, in which case mutants were pre-grown in 2% ethanol/2% glycerol because of the long adaptation time to non-fermentable carbon source conditions. Cells were re-inoculated in described conditions and grown for 4 hours prior to measurements. Mutants were measured at least six times at pH 5.0 and at least three times at all other conditions. Mutants with significantly low pH_c _in any condition are indicated in orange, while mutants with significantly high pH_c _are indicated in blue. Click here for file

Additional file 2 **pH_c _analysis of 432 slow growing mutants**. All strains were grown in standard conditions (2% glucose, pH_ex _of 5.0) and fluorescence was registered in three to six biological replicates, and are presented as average and 95% confidence interval. pH_c _was compared to wild-type (WT) controls in the same replicate, to determine a Z-value. Significance of the pH_c _difference with WT was determined using a two-tailed *t*-test assuming equal variance with a *P*-value < 0.05. ND refers to mutants for which fewer than three replicates were successfully measured. Significantly low pH_c _values are shown in orange, significantly high pH_c _values in blue. Click here for file

Additional file 3 **Classification of mutants**. Mutants are classified as having a growth rate-pH_c _relationship similar to wild type (WT; no significant deviation from the predicted growth rate based on pH_c_-growth rate relationship of the parent strain, low growth rate/pH_c _(significant positive deviation from the parent fit), or high growth rate/pH_c _(significant negative deviation from the parent fit), and are categorized according to functional classification. Click here for file

Additional file 4 **Figures S1 to S4**. See Additional file 5 for further data pertaining to Figure S3. Click here for file

Additional file 5 **Data belonging to the hierarchical cluster plot in Figure S3 in Additional file 4**. Mutants are listed in the order in which they appear in the cluster plot, for all three clusters. Mutant growth profiles were fitted to the parent strain pH_c_-growth rate relationship, and at each time point the Z-value of the digression from the fit was determined compared to the average and variance of 96 parent strain growth curves at the same time point. Time courses during the growth phase (t = 4 h to t = 9 h) of these Z-values were used to statistically categorize the mutants as wild type (WT; 92/173 mutants; 96 parent strain profiles also fall in this category), significantly (corrected *P*-value < 0.01) slow growing (62/173 mutants), or significantly fast growing (19/173 mutants) with respect to pH_c_. Click here for file
