# Supplementary material for: Genome-wide analysis of intracellular pH reveals quantitative control of cell division rate by pHc in Saccharomyces cerevisiae
Source: Genome Biol. 2012 Sep 26;13(9):R80. doi: 10.1186/gb-2012-13-9-r80 (PMC3506951; doi:10.1186/gb-2012-13-9-r80)
Supplement: Additional file 3 — Classification of mutants. Mutants are classified as having a growth rate-pHc relationship similar to wild type (WT; no significant deviation from the predicted growth rate based on pHc-growth rate relationship of the parent strain, low growth rate/pHc (significant positive deviation from the parent fit), or high growth rate/pHc (significant negative deviation from the parent fit), and are categorized according to functional classification. [file gb-2012-13-9-r80-S3.PDF]

Figure S1: Wild type is resistant to changes in extracellular pH. Wild-type was cultivated in unbuffered, pH_{ex} controlled batch fermentors, and titrated to pH_{ex} values indicated using strong acid (HCl). Figures represent pH_c (**A**), growth (**B**), and pH_c plotted against growth rate μ (**C**). Data points represent the average of 12 technical replicates, error bars represent standard deviations.

Figure S2: Hierarchical cluster plots of three main categories of the pH_c-growth rate relationship of each mutant in time. Mutant growth profiles were fitted to the parent strain pH_c-growth rate relationship, and at each time point the Z-value of the digression from the fit was determined compared to the average and standard deviation of the digression of 96 parent strain growth curves at the same time point. Time courses during the growth phase (t=4 h to t=9 h) of these Z-values categorized as WT (92/173 mutants), significantly (corrected p-value <0.01) slow growth (62/173 mutants), or significantly fast growth (19/173 mutants). The three categories were hierarchically clustered using Euclidian complete linkage. Full clustering and fit data can be found in the accompanying Additional file 5.

Figure S3: Slow growing mutants without deviating pH_c show high pH_c/growth rate relationships. Selected slow growing mutants with deletions in genes involved in ribosome biogenesis and central carbon metabolism that do not exhibit aberrant pH_c in our screen were analyzed for pH_c with respect to growth rate. Data bars represent the average z-value over time points t=4 h to t=9 h of curve after comparison to wild-type pH_c/growth arte relationship as described. Error bars represent standard deviation. * p-value < 0.05, ** p-value < 0.01, *** p-value < 0.001.

Figure S4: Other mutants in the inositol pyrophosphate synthesis pathway do not affect pH_c or pH_c signaling to growth rate. pH_c and specific growth rate μ were determined during 16 hour time courses, and μ was plotted as a function of pH_c for parent strain (grey dots), *kcs1Δ* (orange dots), *vip1Δ* (black dots) and *ddp1Δ* (black circles). The data are the aggregate of three biological replicates for each strain.

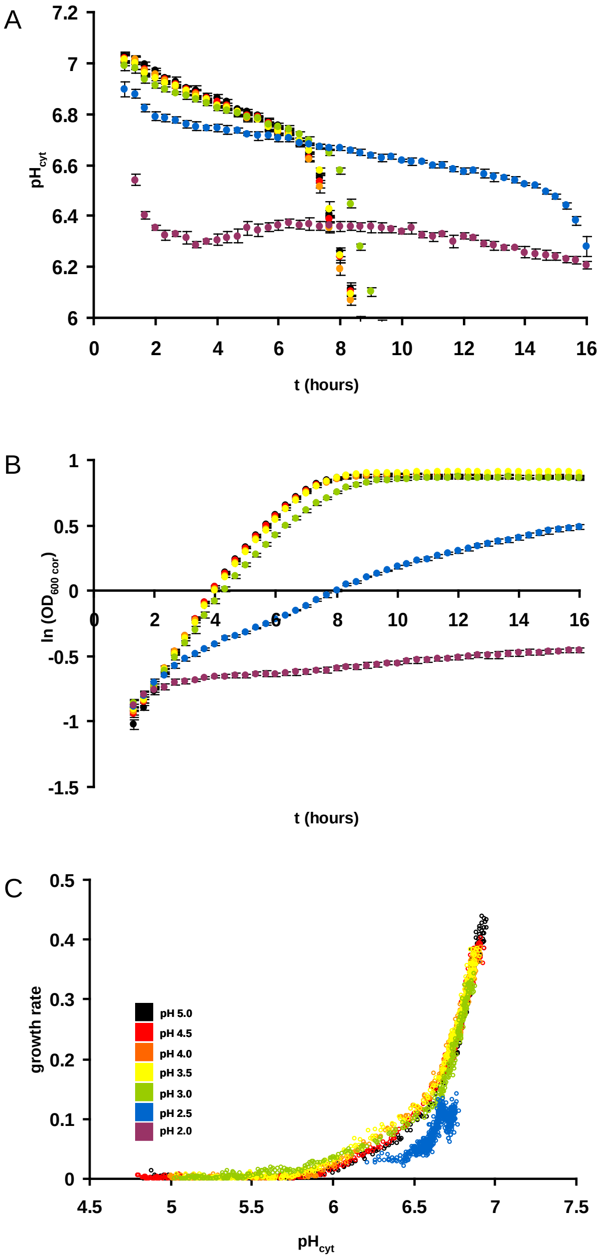
A
pHcyt
t (hours)
B
ln (OD600 cor)
t (hours)
C
growth rate
pHcyt
pH 5.0
pH 4.5
pH 4.0
pH 3.5
pH 3.0
pH 2.5
pH 2.0

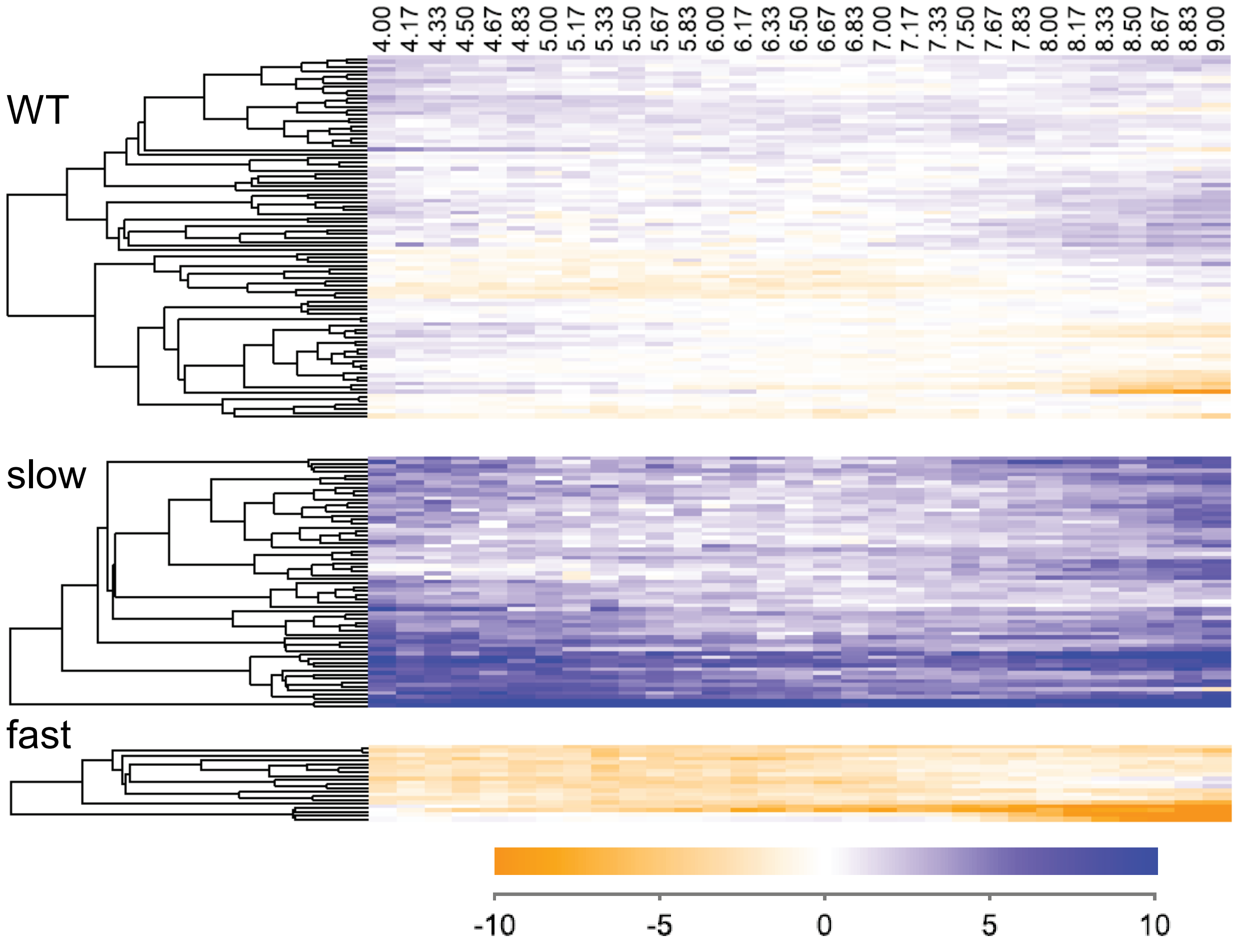
4.00
4.17
4.33
4.50
4.67
4.83
5.00
5.17
5.33
5.50
5.67
5.83
6.00
6.17
6.33
6.50
6.67
6.83
7.00
7.17
7.33
7.50
7.67
7.83
8.00
8.17
8.33
8.50
8.67
8.83
9.00
WT
slow
fast
-10
-5
0
5
10

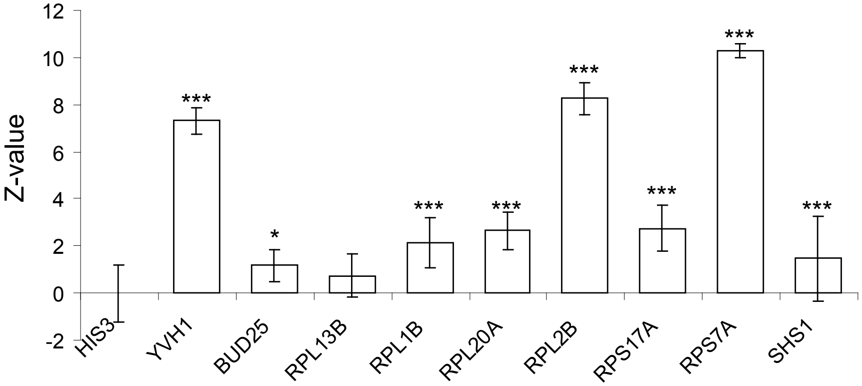

Z-value
12
10
8
6
4
2
0
-2
***
*
***
***
***
***
***
***
HIS3
YVH1
BUD25
RPL13B
RPL1B
RPL20A
RPL2B
RPS17A
RPS7A
SHS1

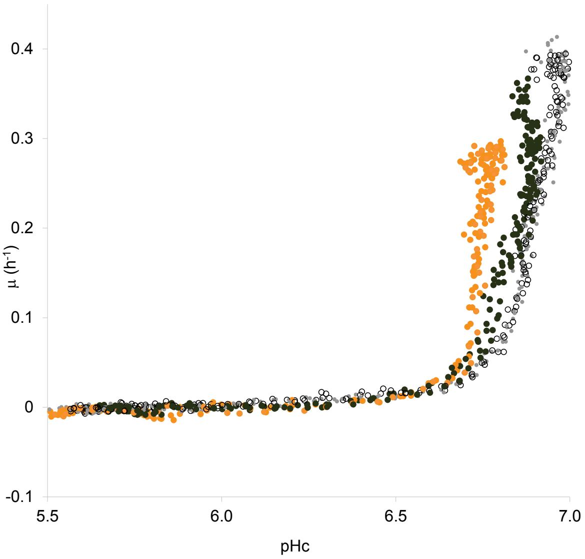
0.4
0.3
0.2
0.1
0
-0.1
μ (h-1)
5.5
6.0
6.5
7.0
pHc
